# Realising the Case Management Ideal in Multi-organisational Coordination Work to Support Work Ability and (Re)employment in Finland

**DOI:** 10.1007/s10926-025-10274-7

**Published:** 2025-02-20

**Authors:** Pirjo Juvonen-Posti, Nina Nevala, Simo Kaleva

**Affiliations:** Finnish Institute of Occupational Health, PO Box 40, 00032 Työterveyslaitos, Helsinki, Finland

**Keywords:** Active labour market policy, Case management, Mixed methods, Re-employment, Return to work, Vocational rehabilitation

## Abstract

**Purpose:**

Inclusivity policies can positively impact labour shortages. The ideal approach to work ability services has shifted from being system centred to being individually tailored and human centred. Service systems are complex in many countries and case managers, referred to here as coordinators, play a key role in achieving the human-centred goal of increasing labour force participation. However, the literature on the practices of these coordinators and their distinct roles in supporting work ability and (re)employment and the contexts in which they do so is limited. This study aimed to clarify what these coordinators do and to explore the extent to which coordinating work meets the ideal of individualised support in different operating contexts.

**Methods:**

The design was a mixed-method study, which utilised two data sources to answer the same complex questions. It consisted of surveys, and in-depth interviews that were based on its results. A total of 241 professionals from various services responded to these surveys, and 15 volunteers were interviewed. The questionnaire data were analysed using descriptive statistical methods, whereas the interviews were examined using thematic analysis. After this, the data were integrated according to a multi-level context framework.

**Results:**

Initially, the coordinators from different sectors shared similar human-oriented values and ways of working. However, crossing administrative and organisational boundaries proved difficult, even though this was essential for the continuity of the client’s process. As a result, ideal case management was not fully achieved.

**Conclusion:**

The findings highlight a necessity for joint policies and practices, and organisational engagement to long-term collaboration.

**Supplementary Information:**

The online version contains supplementary material available at 10.1007/s10926-025-10274-7.

## Introduction

The labour shortage in the EU [1] is already affecting the availability of services and products, compromising both corporate productivity and the stability of national economies. Carefully implemented, disability-inclusive employment policies could help resolve labour shortages by enhancing labour productivity [2, 3] and providing employment opportunities for people with disabilities [4–6].

The work ability services paradigm has shifted from being system centred to being person centred. This underlines the importance of tailoring services to individual needs rather than enforcing a one-size-fits-all model [6, 7]. The current work ability support services in Finland have developed over a long time. In Finland, as in other OECD countries, work ability policies have been reformed to focus on earlier work ability assessments and on integrating health, rehabilitation, social, and employment services. The aim of these reforms has been to address the barriers to job retention, returning to work and re-employment before the problems become so severe that they lead to complete withdrawal from the labour market [8]**.** Coordinated collaboration based on an individual’s rehabilitation needs has also been a key goal of these reforms. Multidisciplinary cooperation has increased in services and rehabilitation for both employed and unemployed individuals, including services that support the return to work of sickness disability benefit recipients [6, 9]. In addition to the efforts to enhance early intervention and coordination, the interactions and services in Finland’s health and employment services have been digitalised on a large scale [10, 11]. In Finland, individuals undergoing vocational rehabilitation have several income options to support them during this period [12]. In many countries, the service system is complex, demanding case management to enhance labour force participation [6]. In numerous Western collaborative contexts, the integration and coordination of various services require significant improvement [4, 13, 14].

Work ability coordination aligns with the principles of case management [5]. Its primary objective is to facilitate cross-agency cooperation and systematic, personalised engagement to help clients achieve their goals of remaining at work, returning to work, or becoming (re)employed, as is the goal of vocational rehabilitation [7]. This involves creating customised sets for clients, integrating services and income support, and tailoring other measures to their specific needs and goals. For this to succeed, interorganisational collaboration within client-specific networks is essential [6, 13]. This in turn requires collaboration with employers, as well as ongoing support for rehabilitation and employment [6]. Professionals in this field may guide supervisors, managers and personnel in public employment and social and healthcare services to help them support employees or jobseekers [13, 15].

The goal to increase the employment of individuals with diminished work ability is hampered by employers still being influenced by various barriers and facilitators when making recruitment decisions. As many employers do not hire people with disabilities [14], employment conditions, and career trajectories remain unequal or unsustainable [16, 17]. Employers harbour several concerns regarding the recruitment and organisational attractiveness of people with disabilities, and employee selection, social integration, and performance management cause challenges throughout the employment cycle [17].

Coordinators are considered pivotal for achieving successful labour participation outcomes [18]. However, the literature on the distinct roles and practices of coordinators and the contexts in which they work is limited. In the context of healthcare, Svärd et al. [19] examined the practices of return-to-work (RTW) coordinators in primary care, orthopaedics, and psychiatry. They found that communication by phone and negotiations varied significantly across these contexts. In workplace settings, Durant et al. [20] identified three crucial tasks that RTW coordinators performed: applying laws, policies, and regulations related to work absences and return to work; contacting the absent worker; and planning the RTW. The RTW coordinators typically had backgrounds in health or occupational health and safety, and this significantly influenced their practices. They primarily collaborated with employees and their supervisors. [20]. In Finland, public employment services (PES) also had work ability coordinators, whose tasks included providing services for jobseekers, training PES staff, helping employers hire people and establishing cooperation networks. A notable difference in their coordination work priorities is that some focus solely on job seekers, while others prioritise employer cooperation [21].

The work of coordinators requires knowledge of applying laws, policies, and social insurance regulations. In addition, interaction, negotiation, and guidance skills are essential when engaging with employees and employers, as is the ability to develop plans and carry out the required follow-ups for individually tailored services that support work ability or RTW initiatives [20, 21]. Coordinators must also be aware of employers’ concerns regarding individuals with disabilities [17]. However, they often lack sufficient knowledge and skills to effectively help employers and employees implement work accommodations [21–23]. Glover and Frounfelker [24] found that employment specialists’ skills significantly influenced the outcomes of individual placement and support (IPS) services. The more successful employment specialists exhibited efficiency, fostered egalitarian relationships with clients, and collaborated effectively with other partners. In contrast, the less successful specialists, although they understood the IPS model, lacked these essential behavioural skills. [24.]

Aligning services and job opportunities with individual needs and preferences in each specific context and situation has been a fundamental aspect of case management, and has been recognised as a key solution in various vocational rehabilitation and employment efforts [21, 25]. The evidence regarding the effectiveness of RTW coordinator models is mixed and inconclusive. According to Kausto et al. [26], an RTW coordinator model may increase employee sickness absence but simultaneously reduce permanent exclusion from the labour market. Conversely, Dol et al. [18] found strong evidence that RTW coordination reduces both the duration of sickness absences and the associated costs. Effective interventions typically incorporate multiple components, such as face-to-face interactions with RTW coordinators, case manager training, an RTW plan, communication and coordination among various stakeholders, and the identification of the barriers to and facilitators of RTW [18]. However, MacEachen et al. [27] concluded that mental health interventions requiring RTW coordinators may take longer than conventional approaches and may not necessarily increase the RTW rate or enhance employees’ RTW self-efficacy. Both MacEachen et al. [27] and Dol et al. [18] emphasise that future studies should be of high quality, that long-term outcomes should be measured and that varied, mixed-method study designs should be used. In the same vein, Holmlund et al. [28] concluded that coordination, the formalisation of coordinators’ qualifications and levels of training, and acknowledgement of the role of organisational factors in the implementation of coordination need to be packaged more clearly.

The literature underscores high expectations of the impact of work ability and (re)employment coordination on individuals’ careers. However, the operationalisation of coordination, especially across various implementation environments, remains inadequately understood. This mixed-method study aimed to investigate how individualised work ability support is realised in diverse settings through professionals’ everyday coordination efforts, viewed through the lens of multi-sector service integration. Our research question was: What factors facilitate or hinder multidisciplinary collaboration in coordination work within a systemic context, both on the organisational level and in client work?

## Materials and Methods

This study was part of a Finnish Government research project (2019–2020) [25]. Its methodological framework was based on a mixed-method study design [29–31].

The integration strategy was in turn based on a joint research mission [29]. The data collected for the survey and interviews were classed under the following seven themes: (1) clients served by professionals engaged in coordination work and their referral processes, (2) types of services and solutions provided and the collaborators involved, (3) the network-building activities of these professionals and their networking partners, (4) the practical coordination of the services and methods used to cross the boundaries of different actors, (5) operations at individual, organisational and network levels, and the tools utilised, (6) the solutions offered to clients, the benefits clients derive from the services, and the pathways the clients follow, and (7) the best practices for supporting job seekers’ employment and enhancing the work ability of those already in employment. The survey asked whether the respondents were willing to participate in an interview.

Before data collection commenced, the researchers collaboratively identified the various stakeholders and their contact persons, using the literature and their professional experience and focusing on the contexts in which the work ability coordinators were employed. We identified a sample of 687 potential participants from among those who had participated in a work ability coordinators training program (*n* = 158) conducted as part of a national project in 2015–2019, which coordinated employment office professionals (*n* = 112), rehabilitation service professionals (*n* = 366) and OHS professionals (*n* = 51) [21, 25]. One researcher was designated to conduct the survey, which was sent to 687 respondents and yielded 241 responses (response rate 35%) (Supplement 1). A total of 72 respondents expressed willingness to participate in an interview.

The survey respondents consisted of 241 professionals (217 women, 24 men), most of whom were aged between 40 and 60, and their work involved supporting work ability and/or employment (Supplement 1). They had diverse educational and professional backgrounds and job titles. The participants worked in different parts of Finland in various parts of the service system, such as social services, healthcare, occupational health services (OHS), PES, educational institutes, human resource (HR) services at workplaces, and in non-profit non-governmental organisations (NGOs). Four out of ten (39%) of the participants had done coordination work for at least ten years.

The qualitative data collection involved interviewing 15 professionals (12 women, 3 men), who were selected from among those (*n* = 72) who had given permission in the survey to be contacted for an interview. Two researchers selected the interviewees so that they represented different respondent groups in terms of their workplaces, duties and parts of the country in which they lived. These interviewees included experts from the PES and the Social Insurance Institution of Finland (Kela), rehabilitation service providers, social and healthcare professionals, OHS personnel, workplace representatives (HR), and NGO actors.

The quantitative survey data collected were categorised on the basis of the joint research themes, and were gathered using the Questback online questionnaire, which consisted of seven themes and 41 questions. The questions used in the survey were formulated on the basis of questions used in earlier studies [6, 21]. The questionnaire was open for four weeks. The study participants were sent an email with a link to the online questionnaire and a letter containing basic information on the study. If they had not responded within the predetermined time slot, they were sent two reminders. The statistical analyses were carried out using the Statistical Analysis Software (SAS) version 9.4. Relative distributions in each question were calculated to describe the data.

The interviews were conducted by two researchers, either via a video conference application (*n* = 12) or by phone (*n* = 3). To minimise interviewer variability, the interview questions were prepared together on the basis of the joint themes. The semi-structured thematic interviews [32, 33] took place between November 2019 and January 2020. Quantitative data were integrated into the interview data collection process: the interviewers had access to the initial descriptive results and the respondents’ survey responses, and they deepened and refined the interviewees’ perspectives and interpretations during the interviews. The interviews lasted 59–89 min and yielded 1079 min (about 18 h) of data. They were recorded and transcribed verbatim by an external professional transcriber using speaker identification, and the researchers verified the transcripts using the recordings. The data consisted of 526 double-spaced pages, and were analysed using thematic analysis [32]. Our thematic analysis highlighted reflexivity, even though we did not strictly follow reflexive thematic analysis [33].

**Fig. 1:**
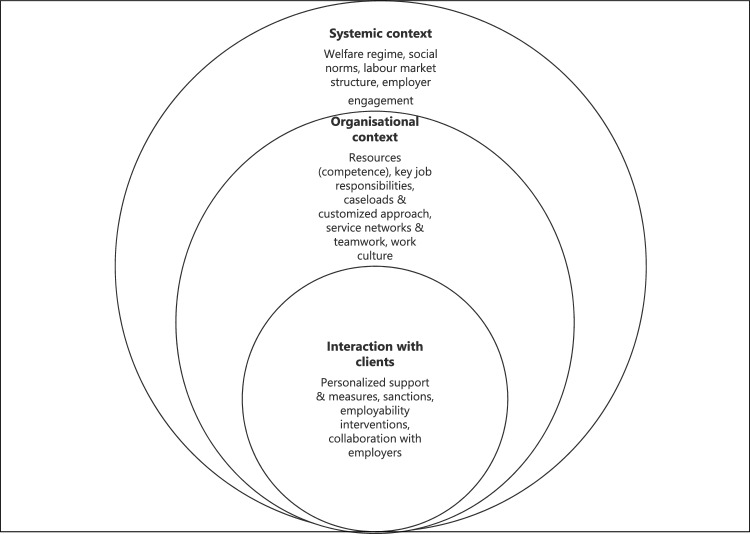
Multi-level context of coordination of work ability and employment support (after [34] (adapted))

The data were further integrated using the theoretical multi-level model proposed by Johnson et al. [34] (Fig. 1). They made a dynamic analysis of the interplay between structure and agency explored the factors that shape an inclusive approach to labour market activation for clients who encounter multiple barriers to work [34]. We found that the client service needs aligned with those described by the work ability coordinators in our data. An organisational, systemic context of activation adopted from Rice was used [35]. Features of a multi-level context of activation casework could also be seen in the preliminary results of our data concerning the coordination of work ability and employment across different sectors, and in multisectoral collaboration. To ensure that the model more accurately reflected the context of our study and to systematise the analysis of the preliminary observations in our data integration phase, we kept the original multi-level context and made only three adaptions to the original model. At the systemic level we added ‘employer engagement’ (1) and at the level of individual client work, we incorporated cooperation with employers (2). We also made clarifications at the organisational level, highlighting competence, a customised approach and teamwork (3).

In the final data integration phase of the analysis, we first drew the conclusions separately, on the basis of both the quantitative and qualitative data, with the quantitative data already partially integrated into the qualitative analysis [36]. The key quantitative results were written into themes, i.e. the quantitative results were transformed into qualitative ones for the purpose of result integration. We then juxtaposed these conclusions in a table created on the basis of the theoretical framework (Fig. 1) [37]. The table used to assist in data integration presented the facilitating and hindering factors at each level, i.e. the quantitative and qualitative facilitating and hindering issues in the systemic and organisational contexts and in the interaction with clients (Table 1). We further categorised the facilitating and hindering factors at each level, in accordance with the theoretical model, into the 14 themes presented in Fig. 1. The integrative analysis proceeded by compiling both quantitative and qualitative data to identify the factors that hindered or facilitated the implementation of coordination work in different contexts. The researchers extensively discussed the results in the data sessions, and throughout the integrative analysis phase.

**Table 1:** Section of the theoretical framework-based table used to compile quantitative and qualitative data in mixed-methods study

Levels and themes	Facilitating factors			Hindering factors		Compiled result
Level: Systemic context	Quantitative results	Qualitative results		Quantitative results	Qualitative results	
Theme: Welfare regime	Network collaboration was most frequently carried out with Kela (82%), the Employment Office (82%), educational institutions and apprenticeship offices (78%), healthcare (77%), and workplaces (70%)		The interviewees described several different types of regional and local multiagency networks, their processes and history	Crossing administrative and organisational boundaries was difficult. It was not possible to track the customer process across these boundaries	The interviewees described the difficulties of crossing administrative and organisational boundaries in detail. This was generally only successful in some regional and long-term local networks	Crossing administrative and organisational boundaries was difficult in coordinating work, even though it was essential for the continuity of the client’s process. In practical client work, this often prevented the tracking of the client process in situations where the coordination of the client’s rehabilitation or employment process continued in another organisation

We present this section of the table as an example of the theoretical framework-based table used to compile quantitative and qualitative data in the mixed-method study. The original table is 6 × 17 (see Fig. 1). In the welfare regime theme within the systemic context of the survey, we found that several actors were active in network collaboration. The interviewees also described several regional and local networks in detail. The hindering factors that emerged in both the survey and interview data were the difficulty in crossing administrative and organisational boundaries. These were compiled as a preliminary result.

## Results

We now report the integrated results concerning the ‘systemic context (SC)’, the ‘organisational context (OC)’, and the ‘interaction with clients (IC)’ (Fig. 1).

### SC Welfare Regime

Crossing administrative and organisational boundaries was challenging in coordination work, even though it was essential for the continuity of the client’s process. This was identified as the most significant coordination barrier in the system. In practical work, this impeded the monitoring of the client process, particularly when the coordination of the client’s rehabilitation or employment process transitioned to another organisation. Accessing client information that supported multi-administrative work was difficult or impossible:

…it always bothers me when it comes to managing clients ... when the organisational boundaries are so high, and each one has their own system that others can’t peek into… the [national patient information system] service that we have here in healthcare now helps a bit … we can see some basic healthcare information... the main thing should be the work... it’s a bit tough in the sense that communication … [I9, social and healthcare]

...when the assignment comes to us from the pension insurance company … we don’t inform OHS about the progress of the process in any way... when the client comes to us for an appointment... we ... write that [as] a guidance visit related to vocational rehabilitation, we encrypt the entry ... it doesn’t transfer to the national [national patient information] system either... So when the [client’s] doctor opens their [OHS]database, our work ability coaching visits ... are not visible there.”[I1, OHS]

### SC Social Norms

Integrated and shared social norms were lacking at several administrative levels**.** A hindering factor in multisectoral coordination work was the lack of norms defining who is responsible for recording information, what information should be recorded, and where it should be documented. The only facilitating exception was that the service was tailored for long-term unemployed jobseekers with disabilities, known as Employment-Promoting Multidisciplinary Cooperation (TYP). This joint operating model, involving municipalities and officials from Kela and PES, assesses, plans and manages the clients’ employment process collaboratively, using a process-responsible approach. However, the professionals often found themselves recording the same information twice: first in the sector system and then again in the shared system.

The role of social norms also arose in client-based decision-making. A hindering factor described was that institutional decision-making tended to predominantly rely on the documents produced by the professionals, which potentially overrode the client’s desires and goals.

“... the goal is ... clear, and it’s also defined by commission of the pension company. … during [client’s] visits, we’ve generally been able to assess what kind of commission is coming from there, whether it’s just a work trial or retraining or something else. But ... if the pension company has set very specific limits to what they’ll cover, we can try to justify it if we see that it’s not sufficient action, but … the goal setting comes from there... certain limitations will inevitably come from there.” [I1, OHS]

### SC Labour Market Structure

The PES participants felt that they were best positioned to monitor the changes in the labour market and the labour needs. In the survey, the majority (82%) of the professionals reported working in multidisciplinary collaboration with employment services. The PES coordinators were able to share their expertise and best practices concerning employment and work ability support within their national and regional networks.

### SC Employer Engagement

To demonstrate their commitment to supporting work ability and minimising work disability costs, some employers have integrated work ability coordinators into their HR departments or included these services in their OHS agreements. In large companies, even a single work ability coordinator can be a valuable resource, as their presence facilitates easy discussions on work ability between employees and supervisors. However, the coordinators who work with OHS providers are guided by company agreements and legislation, which impose limits on the number of guidance sessions and can sometimes hinder the coordination process.

“… clients tend to contact me quite easily, as we have a little over [500–1000] employees ... the threshold for coming to me is quite low. People either reach out to me themselves or their supervisor contacts me, or they come to me via OHS. [I 14, employer/HR]

Employers’ attitudes towards hiring people with disabilities varied. Some employers were only interested in offering work trial periods, during vocational rehabilitation, for example. However, some employers were willing to hire, provided their experience of the employee’s performance during the work trial period was positive.

“… [some employers]...open-minded and ready to consider the possibility of employment … as a paid position…but… many employers are not willing to cooperate. [...] our approach with the [clients] is that once the skills assessment is done …everyone starts looking for suitable employers themselves... and the goal is always to submit at least five applications for work trials …” [I4, vocational rehabilitation]

The OCs were public social- and healthcare services, private OHS, PES, educational institutes, HR services in private and public workplaces and NGOs.

### OC Resources (Competence)

**Fig. 2:**
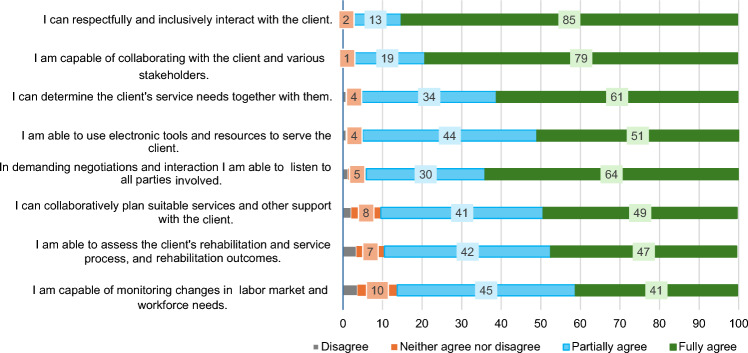
Responses of those engaged in coordination work (*n* = 241) to statements about their competence in coordinating employment and work ability support (percentage of respondents)

The professionals rated their skills highly: 85% felt confident in inclusive client interactions, 79% when collaborating with clients and stakeholders, and 64% when listening during challenging negotiations. Fifty-nine per cent felt capable of guiding clients through the service system and assessing their needs. They felt least confident when monitoring labour market changes (Fig. 2).

To summarise, on the systemic context level, crossing organisational boundaries is crucial for the coordination of work ability and (re)employment, especially when client processes such as rehabilitation or employment transition from one organisation to another. However, crossing these boundaries is difficult. Poor system integration, communication gaps, a lack of unified social norms—regarding information recording, for example—makes accessing client information across systems difficult and hinders coordination and process continuity, but we found one positive exception. Decision-making can sometimes favour institutional documents over client preferences. The only party that was able to follow clients’ paths was the PES, as they were able to monitor employment statuses. Employer engagement in supporting work ability varied; some integrated work ability coordinators into their HR or OHS, but guidance sessions may still be limited by agreements and legislation. Attitudes towards hiring people with disabilities were mixed: some employers were open to hiring after successful work trials, whereas others only offered trial periods.

### OC Coordinators’ Key Job Responsibilities

Coordination was often part of only professionals’ roles, and client work was typically conducted independently without immediate multidisciplinary team support. However, for most of the interviewees, coordination was a primary or integral duty, and involved identifying service needs, facilitating cooperation and selecting methods for supporting work ability. Although most professionals met clients at their premises, a quarter met them at their workplaces. A significant hindrance was the lack of systematic documentation; some professionals did not document their work at all.

### OC Caseloads, Opportunities for a Customised Approach Tailored to Each Client

The coordinators’ client loads varied within the service system**.** Although client-centeredness was a key principle, opportunities to tailor coordination to individual needs and goals were limited. The professionals and their organisations were satisfied with the collaboration in local or regional networks, but found it time consuming or difficult in other contexts**.**

### OC Service Network and Teamwork

**Fig. 3:**
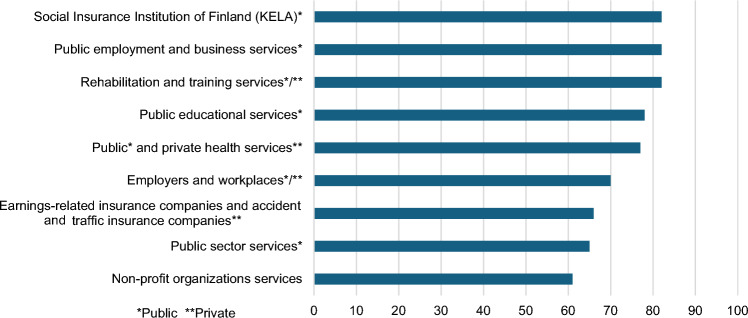
Cooperation partners in coordination work to support clients’ employment or continued employment over the last year (*n* = 241, several response options, percentage of respondents, *Public **Private)

Collaboration was most commonly with Kela (82%), PES (82%), educational institutions and apprenticeship offices (78%), healthcare (77%), and workplaces (70%) (Fig. 3).

Networks and teams varied locally, by region and by organisation. Sometimes they were built and led on a temporary basis by a project, for example, but several quite steady networks maintained by two or three different entities were described. They often had no formal status in any organisation but were crucial for ensuring that the client received the required service.

“… the work ability coordinators within [the same administrative sector] network are ...significant ... for this area, two or even three quite similar… local networks have emerged ... the [CITY’s] work ability [network]... [also] throughout the [REGION] ... and [one] here in the [AREA’s] hospital district’s ... project ... created this kind of work ability coaches’ network for this [REGION] ... there’s a need for them. ... with the [various organisations’ projects], a working model has emerged in which we organise these intermediary labour market workshops, ... through ... these intermediary labour market operators, in some cases, contact me directly...” [I6, PES]

The concept of multi-professionalism was commonly implemented through two primary methods. First, the professionals typically operated independently while working with their clients, later collaborating and networking with other professionals and workplace operators. Second, multi-professional work also involved addressing clients’ concerns as part of a multidisciplinary team. In the regional and local networks, the shared understanding of each process and the level of agreement on roles and tasks varied and required joint development. The coordinators wished for a clearer definition of networking as part of their job description. Although multidisciplinary collaboration was considered important in coordination work, the respondents wanted the interaction within it to progress from sector-specific problem definition to integrated solutions.

“...network work is kind of … challenging but then it’s also fruitful because it enables the creation of a common vision ... it’s always problematic if we start, in a network meeting ... to determine what this client’s problem is. When [social work] defines it then (-) the employment office then (-) the social insurance institution then (-) healthcare, well, you get as many definitions of the problem as is possible (--). It’d be better to ask what each of us can do within the substance framework, so that the client’s situation, you know, improves. “ [I10, TYP]

### OC Work Culture

The work culture, service processes, and collaboration methods of the coordination work varied across regions, and different combinations produced either smooth cooperation and clients’ processes or great difficulties, at worst even halting the client’s rehabilitation process. For instance, all working-age individuals’ work-related issues were not universally addressed in social and healthcare services. Long-term client guidance often relied on collaborative practices that crossed administrative and organisational boundaries. Some workplaces engaged in effective collaboration with OHS; however, guidance and coordination of support for work ability and rehabilitation outside the workplace were limited, except for collaboration with the employer’s pension institution. As described earlier, crossing administrative and organisational boundaries was frequently challenging in practice. The professionals involved in the coordination described approaching clients from their own professional perspectives, emphasising respectful yet relaxed interactions, attentive listening and client empowerment.

To summarise, on the organisational context level, coordination was often part of the professionals’ broader roles, and much client work was done independently. For many, coordination was a primary duty and focused on service needs, cooperation, and supporting work ability. Although most professionals met clients at their premises, a quarter did so at their workplaces. A major issue was the lack of systematic documentation. Client loads varied, and although client-centeredness was a key principle, tailoring services to individual needs was limited. Collaboration, with was commonly with Kela, PES, educational institutions, healthcare, and workplaces, was generally satisfactory, but could be time consuming. Networks varied in structure but were essential for client services. Multi-professionalism involved professionals working independently and later collaborating or addressing client concerns in multidisciplinary teams. The coordinators sought clearer networking roles and desired integrated solutions. Work culture and processes varied by region, which affected the smoothness of client processes. OHS collaboration was deemed effective in some workplaces, but broader work ability coordination was limited. Crossing administrative boundaries was challenging, and professionals focused on respectful, empowering interactions.

### IC Personalised Support and Measures

**Fig. 4:**
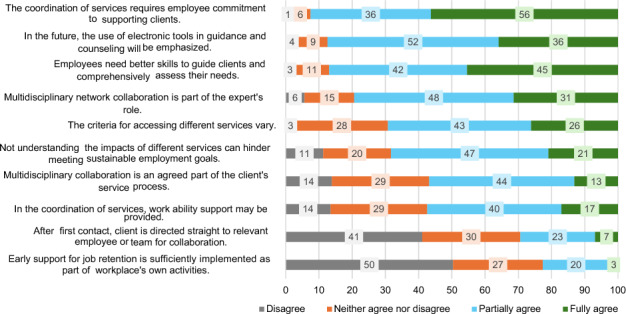
Responses of those engaged in coordination work (*n *= 241) to first statements on support for work ability and coordination of services (percentage of respondents)

A human-centred, individualised approach was an important starting point for all. Clients were often referred to coordination services to prepare various plans. Based on the data, the service had, already before the COVID-19 era, mainly been electronic or provided via the phone and this was expected to increase. According to the professionals, clients were not able to find the services they needed without their personal guidance. The duration of the multidisciplinary services varied greatly, averaging from three to six months. Most of the professionals (92%) agreed that the coordination of services required employees to commit to client goals (Fig. 4).

In most cases (65%), the professionals had served 1–30 clients in the last month. Typically, the professional and the client met one to nine times (70%). Most respondents indicated that the meetings usually occurred at their own premises, either alone (87%), with a multidisciplinary group (44%), or with a work partner (36%). In addition, client processes sometimes continued informally, outside administrative records.

“… they are our clients [administratively]... even if their services are elsewhere, they want us to be aware, … [the PES coordinator] is … a support and safety net for the client ... a person who stays informed about different services and records these matters.... like service guidance... The client can convey information by email, via the internet or by calling. It …depends on … how they want to handle things“[I11, PES]

The respondents emphasised the importance of client participation in all the process stages and in decision-making. The professionals reported that the clients could help define objectives (96%) and select measures (89%). A significant majority (66%) disagreed and claimed that clients typically did not attend the meetings in which their issues were discussed. Despite these principles, the clients often followed uniform service paths due to limited resources for tailoring the services. Coordination efforts aimed to build client goals through participatory means and innovative solutions. Those involved in coordination reported extensive efforts to manage services and benefits.

**Fig. 5:**
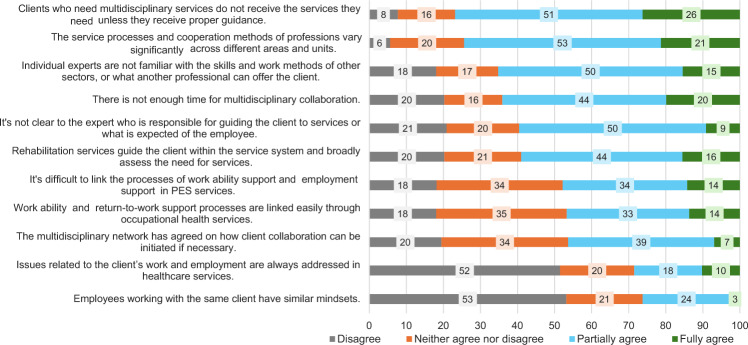
Responses of those engaged in coordination work (*n* = 241) to second statements on support of work ability and coordination of services (percentage of respondents)

The professionals believed that their actions were crucial for supporting clients’ work ability or employment, with 78% agreeing that clients needing multidisciplinary services would not receive them without guidance (Fig. 5). However, they struggled with systematic and long-term monitoring of client processes due to fragmented efforts and a lack of clear instructions or tools. The coordination professionals at PES could monitor client employment status dichotomously (‘employed’ or ‘not employed’). Otherwise, progress was tracked through follow-up appointments. Workplace coordinators monitored clients’ job retention, whereas social and healthcare professionals used personal diaries to track clients’ statuses. The service processes and cooperation methods were diverse, and professionals were often unaware of other sectors’ expertise and services. Despite sharing the principles of human-oriented support for work ability and employment, over half of the respondents disagreed with the claim that professionals working with the same client had similar perspectives or that employment-related issues were addressed in healthcare services.

### IC Employability Interventions

Service network cooperation was prevalent in PES (82%). The coordinators talked in detail about creating plans. PES and TYP coordinators described long, sometimes years-long, vocational rehabilitation processes. Job-seeking was also described.

### IC Collaboration with Employers and Supervisors

This outcome was controversial: according to the survey, 70% of the professionals had collaborated with the workplace during the last year, but based on the interview data, workplace collaboration was often limited or non-existent. When a coordinator worked in either HR or OHS, workplace collaboration was possible within the network of the respective employer. Matching the job to the person’s work ability was difficult because the coordinators most often met clients on their own premises, so did not get to see the client’s work and work environment factors.

To summarise, on the client interaction level, a human-centred approach was vital for individualised support, and services were mainly provided electronically or via the phone, even pre-COVID. Personal guidance was crucial for clients to find the services they required, and the services lasted for three to six months on average. Coordination required commitment to client goals, and most professionals met their clients one to nine times, often at their own premises. Client participation in decision-making was emphasised, but due to limited resources, uniform service paths were common. Systematic monitoring was challenging due to fragmented efforts and a lack of tools. PES had strong service network cooperation (82%), and coordinators created detailed plans. Long vocational rehabilitation processes and job-seeking were common in employability interventions. Although 70% reported workplace collaboration, this was often limited. Collaboration was more feasible when coordinators worked in HR or OHS. Matching jobs to work ability was difficult, as meetings usually took place outside the workplace.

## Discussion

Crossing administrative and organisational boundaries is difficult in coordination work, as described earlier [4, 13, 25]. The greatest obstacle to coordination was identified as a systemic issue that manifested across diverse organisational contexts and affected client work (Fig. 1). Crossing the boundaries of organisations to ensure that the coordination for the client’s re-employment or rehabilitation process continues is a key part of ideal case management [5] and an important mechanism for promoting employment [18], but this seldom happened. Instead, the organisational boundaries seemed to be insurmountable. Also, a lack of integrated and shared social norms across multiple administrative levels was evident. Responsibilities were unclear, and some individuals made their work unattainable or did not even document it. It is difficult to evaluate in hindsight how the clients’ rights and goals were achieved in such processes.

In everyday client work, the boundary-crossing challenges were addressed through network and multi-professional teamwork. Despite this being crucial for coordination, these teams and networks often functioned informally, lacking jointly agreed-upon roles, processes and documentation procedures. Controlling one’s caseload and cross-cutting, short-term support were seen as solutions to the problem of crossing organisational boundaries. As Johnson et al. [34] have also noted, the responsibility for coordination frequently fell on a single coordinator operating within their unique multidisciplinary context.

Collaboration was most common between social insurance, public employment, healthcare, educational institutions and apprenticeship offices and workplaces. When the respondents described their collaborative networking, they only occasionally mentioned academies and workplaces. It seems that workplace collaboration was difficult for some to realise, and for those working at workplaces, it was also difficult to pass on the client and their issues to other networks. Getting professionals to pay attention to a person’s work and career can be challenging, because it requires a substantial paradigm shift [38]. It may be easier for public coordinators to choose public services for their client than to begin matching the job to the person with workplace actors and vice versa. The coordinators, even during brief interactions, received positive feedback from clients for their ability to grasp the overall situation. They also positively assessed their own key professional skills. However, they lacked familiarity with the skills, working methods, and contributions of professionals in other sectors. This lack of clarity among the experts responsible for referring clients is consistent with findings from previous studies [21, 26].

As regards the novelty of our study, its data consisted of coordination workers who operated in various parts of the service system. Earlier reports have mainly concerned RTW coordinators. The main methodological strength of this study was its integrative mixed-method design, and that it utilised different data sources to answer the same complex research questions, which made it possible to continue the analysis by further integrating these meta-inferences [29, 37]. We sought a comprehensive overview of coordination at the system, organisation and client work levels, and the same complex research question enabled iteration during the analysis; it increased the credibility and reliability of our research, and also enabled the detection of context-related exceptions.

As the topic has scarcely been studied, only a few validated questions were available for us to use. The survey’s response rate was moderate (35%) despite the several measures we took to increase and guarantee a good response rate. Statistics Finland [39] has been concerned about the significantly decreasing response rates in Finland. The participants were mostly women, which is in line with other studies of work ability coordinators [21] or rehabilitation professionals in Finland [6]. Thus, this sample can be considered representative. Our results can be considered reliable, as the respondents consisted of experienced professionals of different ages operating in different parts of the country from the public, and private sectors as well as NGOs.

Unfortunately, we were unable to interview everyone who was willing, and another weakness here is that, because we tried to get interviewees from different sectors and from geographically different parts of the country, the group we selected to be interviewed was well experienced in this work. However, this brought reflexivity to the interview material.

The study design utilised different data sources to address the same complex questions through concurrent, dependent data collection [29]. A mixed-method approach was applied because it enabled the combination of quantitative and qualitative research approaches and data, particularly inference techniques, to facilitate a deeper understanding of the corroboration [40] and integrative strategies in the analysis [41]. Triangulation [31] and inter-method mixing [42] were employed to gain an in-depth understanding of the complex [43], multi-faceted nature and constructs of case management in different settings, as well as its outcomes. The integration of quantitative and qualitative data was facilitated by having the same themes in the survey and interviews. The chosen theoretical model [34] was suitable for the integration and enabled us to jointly restructure the data. However, we had no data on sanctions, and although we had participants from different geographical areas, we were unable to obtain any specific results concerning labour market structure.

The study reveals that crossing administrative and organisational boundaries is challenging for coordinators, even though it is crucial for the client’s situation. Future efforts should develop methods to facilitate boundary-crossing based on client needs, and include common tools for recording and monitoring client information. Systematic monitoring would provide valuable feedback for professionals. Building well-functioning regional networks with clearly defined roles and responsibilities is also essential. Networking would benefit both clients and professionals. Another need is competent understanding of the roles of other actors, including employers, and increased visibility of PES and the rehabilitation services in social and healthcare sectors. More information is needed on income options during rehabilitation and the use of electronic tools should be intensified. Multidisciplinary cooperation benefits from joint training of professionals from different fields, so that a common language and understanding of the service system can be created.

Future research should clearly define the roles and strategies of work ability and employment coordinators (see [27]) and address the impact on employees’ quality of life in addition to job retention and employment outcomes (see [18, 27]) on employer engagement in inclusive recruitment. Moreover, instead of high, inaccurate outcome hopes (see [34]), coordination should have detailed packaging. Coordinators’ qualifications and training levels need to be formalised and the role of organisational factors in the implementation of coordination acknowledged (see [28]). There is a clear need for well-designed, multi-perspective, mixed-method studies.

## Conclusions

As described, case management, here the coordination of work ability and (re)employment, is either an independent entity or a part of vocational rehabilitation and employment support services, depending on the country. Therefore, our results have significance beyond Finland, for example, for strengthening client cooperation, which has been identified as an important mechanism in multisectoral and workplace collaboration, i.e. in enhancing sustainable employment.

To achieve optimal case management, it is essential that we address the systemic barriers that impede effective coordination. Overcoming these obstacles can improve labour force participation and work ability. However, it requires rethinking policies, organisational structures and developing integrated service systems, as well as organisations’ commitment to promoting cross-organisational collaboration, and the engagement of employers and workplace actors. Establishing frameworks for smoother transitions and client support continuity is also crucial, whether this be through digital or other means. Coordinators need training, support and resources to effectively navigate and bridge administrative and organisational boundaries. Further research should explore specific strategies and tools for overcoming coordination challenges in different contexts.

## Supplementary Information

Below is the link to the electronic supplementary material.

Supplementary file1 (DOCX 16 KB)

## Data Availability

No datasets were generated or analysed during the current study.
