# Supplementary material for: Realising the Case Management Ideal in Multi-organisational Coordination Work to Support Work Ability and (Re)employment in Finland
Source: J Occup Rehabil. 2025 Feb 20;36(2):394–406. doi: 10.1007/s10926-025-10274-7 (PMC13100016; doi:10.1007/s10926-025-10274-7)
Supplement: Supplementary file 1 — Supplementary file1 (DOCX 16 KB) [file 10926_2025_10274_MOESM1_ESM.docx]

**Realising the case management ideal in multi-organisational coordination work to support work ability and (re)employment in Finland**

**Supplement 1** Background factors of participants in coordination work (n=241) in survey

| Variable |  | N | % |
| --- | --- | --- | --- |
| Gender | Women | 217 | 90 |
|  | Men | 24 | 10 |
| Age, years | <40 | 51 | 21 |
|  | 40–59 | 166 | 70 |
|  | > 60 | 24 | 10 |
| Education | Comprehensive or general upper secondary education | 24 | 9 |
|  | Vocational education (school, college-level qualification, vocational qualification) | 47 | 20 |
|  | Academic degree | 205 | 85 |
| Work experience of work ability support, years | <10 | 144 | 61 |
|  | 10–19 | 60 | 25 |
|  | >20 | 32 | 14 |
| Employer | Public sector | 100 | 41 |
|  | Private sector | 114 | 47 |
|  | Third sector | 27 | 11 |
| Size of workplace | Micro (<10 employees) | 6 | 2 |
|  | Small or middle-sized (10–249 employees) | 112 | 47 |
|  | Large (> 250 employees) | 106 | 44 |
|  | Not known | 17 | 7 |
